# Supplementary material for: ‘It is not fashionable to suffer nowadays’: Community motivations to repeatedly participate in outreach HIV testing indicate UHC potential in Tanzania
Source: PLoS One. 2021 Dec 22;16(12):e0261408. doi: 10.1371/journal.pone.0261408 (PMC8694479; doi:10.1371/journal.pone.0261408)
Supplement: S1 File — (ZIP) [file pone.0261408.s001.zip › Tools/FGD with CommunityLeaders version 2.docx]

**Community Leaders for Communities around the 4 HUBS**

**Introduction**

- We are social scientists who work with a project that tries to find out whether it is possible to provide decentralized care to people living with HIV in Shinyanga and Simiyu region. The project has two components: to test as many people as possible in order to identify HIV-positive people, to link these to care as soon as possible, without waiting for the immune system to deteriorate and to provide care such as the distribution of ART and check-ups in community Clubs
- The project is run by the Archdiocese of Shinyanga and CUAMM but with the permission of the Tanzanian government. At the moment you may have seen CUAMM running testing campaigns in the community
- Our role as social scientists is to advice the project on how to adapt their procedures to match to what community members desire and to involve the voice of the community leaders and members as much as possible. Therefore we have asked for a discussion with you.

**Introduction**

- Background - Community leaders
  - What are their roles in the community
  - How do they see their roles in guiding the community on health related matters
- Main issues in the community
  - in relation to health,
  - socio-economic issues
  - youth, old age etc
- Leadership and organisation in the community
  - Besides yourself, who are other influential people in the community, especially related to health issues?
  - What are regular functions in which the community comes together (baraza etc)
  - What community groups exist here (savings and loans, women’s group, any men’s group, any youth groups, such as soccer teams etc)
- History of HIV in the community
  - Do you consider HIV to be a problem in this community?
  - Amongst which groups?
  - What responses have there been to HIV in your community in the past

Is there currently any stigma, can you give examples?

- - Are there also role models?
  - Are there other health programmes addressing HIV in this community?

**Test and Treat experience**

- Awareness and opinion of HIV testing
  - What do you know about current HIV testing? (what are the possibilities)
  - Before we introduced ourselves, had you heard of early testing and treatment? If no explain in more detail (You start treatment immediately with two advantages: 1) you have a better health outcome. 2) you are no longer as infectious if you take treatment well.
  - What is your opinion on this change?
    - Probe: what do you think of the idea that you test as many people as possible?
    - Probe: What do you think of the idea that you start people on treatment immediately
  - We all know not everyone wants to go for testing. We are interested in finding out which groups these are. Would you have an idea?
  - Right now the project is doing outreach campaigns in the hopes to reach this groups: do you think this is an effective strategy (Probe why or why not)
  - Would you suggest other strategies to reach these groups? (probe: influential others)
  - What could be strategies to convince people of the importance of early testing that the health facilities could offer? (information meetings, barazas, community dialogues, materials, involving religious leaders, traditional healers etc)
- **Current HIV care**
  - What do you know about current HIV care? (adherence etc)
  - The project proposes decentralized care: have you heard of this? (explain clubs model: clients who are adherent for six+ months receive treatment no longer at the health facility but at community based clubs, to facilitate access
  - What do you think about this change?
  - How would these clubs need to be organised to be acceptable?
- **Test and Treat talk in the community (do you hear people talking about EAAA, what and who are they talking about, give an example, any rumors)**
- **Test and Treat & community leadership** (would the community leadership like to do anything related to Test & Treat
- **Test and Treat & Traditional healers** (Do you think traditional healers can do anything related to Test and Treat?, if so how?).
