# Supplementary material for: ‘It is not fashionable to suffer nowadays’: Community motivations to repeatedly participate in outreach HIV testing indicate UHC potential in Tanzania
Source: PLoS One. 2021 Dec 22;16(12):e0261408. doi: 10.1371/journal.pone.0261408 (PMC8694479; doi:10.1371/journal.pone.0261408)
Supplement: S1 File — (ZIP) [file pone.0261408.s001.zip › Tools/FINAL_IDI_LTFU_En.docx]

**IDI LOST-TO-FOLLOW-UP CLIENTS**

1. **RESEARCH QUESTION**

How many clients who are enrolled through CBT and CTC testing under test and treat are lost to follow up within 6 months after treatment initiation

What are the reasons for disengaging in chronic care by patients who initiated care under the Tanzanian Test and Treat Policy

1. **OBJECTIVES**

The study seeks to:

1. Ascertain the proportion of patients who are truly lost to follow up among those enrolled in the T&T study
2. Assess the point (in months from time of ART initiation) when patients are likely to be lost to follow up
3. Describe the reasons for loss to follow up from patients’ and/or primary caregivers’ perspective of patients enrolled in the Test and Treat study

**OPERATIONAL DEFINITION**

| **TERMINOLOGY** | **OPERATIONAL DEFINITION** |
| --- | --- |
| **Loss to follow up** | Refers to not keeping ART refill appointment for a period of 90 days or longer from the last booked refill appointment date, yet not classified in patient clinical outcome as ‘dead’ or ‘transferred-out’ (MoH def check). In this study LFTU refers to any person who misses a scheduled clinical visit for 90 days from the last scheduled date. |

**QUANTITATIVE**

**SECTION A:** **SOCIO-DEMOGRAPHIC CHARACTERISTICS**

| **Ques. Seq.** | **QUESTION** | **RESPONSES** | **CODE** | **SKIP** |
| --- | --- | --- | --- | --- |
| **A1** | **Age of participant** | 18-24  25-29  30-34  35-39  40-44  45-49  ≥50 | 1  2  3  4  5  6  7 |  |
| **A2** | **Specific age in years** | ----------years completed |  |  |
| **A3** | **Sex** | Male  Female | 1  2 |  |
| **A4** | **Residence** | Urban  Rural | 1  2 |  |
| **A5** | **Marital Status** | Never married  Married  Separated  Divorced  Widowed | 1  2  3  4  5 |  |
| **A6** | **Highest level of education attained** | No education  Primary  Secondary  High school  Tertiary | 1  2  3  4  5 |  |
| **A7** | **Religion** | Christianity  Muslim  Traditional  Non believer  Other (specify) | 1  2  3  4  5 |  |
| **A8** | **Employment status** | Not employed  self employed  working part-time  working full time | 1  2  3  4 |  |
| **A9** | **Monthly household income** | E0-1000  E1001-5000  E5001-10,000  10,001-20,000  +E20, 001 | 1  2  3  4  5 |  |
| **A10** | **Distance to nearest ART Clinic** | 0-5 kilometres  6-10 kilometres  11-20 kilometres  More than 20 kilometres | 1  2  3  4 |  |
| **A11** | **HIV disclosure status** | Disclosed  Partially disclosed  Not disclosed  Don’t know | 1  2  3  99 |  |
| **A12** | **Has treatment supporter** | Yes  No  Not sure | 2  1  99 | ***If no or not sure, skip to section B*** |
| **A13** | **Relationship with treatment supporter?** | Wife  Husband  Parent  Sister  Aunt  CHV/Worker  Other (specify)  No relationship | 1  2  3  4  5  6  7  99 |  |

**SECTION B: EXPERIENCE WITH HIV TESTING**

| **Ques. Seq.** | **QUESTION** | **RESPONSES** | **CODE** | **SKIP** |
| --- | --- | --- | --- | --- |
| **B1** | **Date tested for HIV** | Yes  No  Don’t remember /no stated in chronic care file | 1  2  99 |  |
| **B2** | **HIV test done at** | Hospital  Health centre  Public Health Unit  Clinic  Outreach  Chemist/pharmacy  Other (specify | 1  2  3  4  5  6  88 |  |
| **B3** | **Counseled prior to HIV test** | Yes  No  Don’t know/not stated in chronic care file | 1  2  99 | ***If no or don’t remember, skip to section C*** |
| **B4** | **Counseled post-HIV test** | Yes  No  Don’t know/not stated in chronic care file | 1  2  99 |  |
| **B5** | Counseled by | Expert client  Nurse  Dr  Other (specify) | 1  2  3  4 |  |

**SECTION C: CLINICAL CHARACTERISTICS AT BASELINE**

| **Ques. Seq.** | **QUESTION** | **RESPONSES** | **CODE** | **SKIP/COMMENT** |
| --- | --- | --- | --- | --- |
| **B1** | **Body weight** | <40kg  40-49kg  50-59kg  60-69kg  +70kg | 1  2  3  4  5 |  |
| **B2** | **WHO Clinical stage** | I  II  III  IV  Don’t know/not available in chronic care file | 1  2  3  4  99 |  |
|  | **CD4 cell count test** | Done results available in chronic care file  Done results not available in chronic care  Never done  Don’t know/not available in chronic care file | 1  2  3  99 |  |
| B3 | **CD4 cell count** | <50  50-200  201-350  351-500  +501  Don’t know**/**not available in chronic care file | 1  2  3  4  5  99 |  |
| B4 | **Viral load** | Done results available in chronic care file  Done results not available in chronic care  Never done  Don’t know**/**not available in chronic care file | 1  2  3  99 |  |
|  | **Functional status** | Ambulatory  Bedridden | 1  2  3  4 |  |
|  | **Clinical presentation on last visit** | CD4 cell count | --------------- | Write figure of last results |
|  |  | Viral load | --------------- | Write figure of last results |
|  |  | WHO clinical stage | --------------- | Write figure of last results |
|  |  | Functional status | ---------------- | Write figure of last recorded status |

**SECTION D: EXPERIENCE WITH ART**

| **D1** | **Period on ART in months** | 0-1 month  1-3 months  3-6 months  6-12 months  13-24 months | 1  2  3  4  5 |  |
| --- | --- | --- | --- | --- |
|  | **Pre-ART counseling provided** | Yes  No  Don’t remember /no stated in chronic care file | 1  2  99 |  |
| **D2** | **ART Regimen** | 1^st^ line  2^nd^ line  3^rd^ line | 1  2  3 |  |
| **D3** | **Side effects** | Yes  No | 1  0 |  |
| **D4** | **Adherence** | Optimal  Average  Poor | 1  2  3 |  |
| **D5** | Reasons for suboptimal or poor adherence |  |  | Write reason(s) as recorded in chronic care file |
| **D6** | Patient outcome | Alive but relocated (t/f out)  Alive and dropped out of care  Dead  Other  Not stated in chronic care file | 1  2  3  4  99 |  |
| **D7** | Reasons for loss to follow up |  |  | Write reason(s) as recorded in chronic care file |

**DETERMINANTS OF, AND REASONS FOR LOSS TO FOLLOW UP OF PATIENTS ENROLLED IN EARLY ACCESS TO ART FOR ALL (EAAA) STUDY IN HHOHHO REGION, SWAZILAND**

**QUALITATIVE INTERVIEW GUIDE**

***Experience of HIV testing and positive diagnosis***

1. Please tell me about the day when you were tested for HIV, from the time you were tested, to when you entered the facility and when you left the facility? (probe for month, year, testing place, facility, experience of being tested)
2. Was this your first test and what was the reason for getting an HIV test?
3. Did anyone influence your decision you talk to anyone prior about getting tested? Who did you talk to and why or why not? (probe for family, partner, in-laws, friends, TH, CHW others)
4. Did anyone influence your decision to get tested?
5. What did it mean to you being HIV positive?
6. What was your reaction to the HIV positive diagnosis?
7. Did you discuss or share your HIV status with someone? Why, why not? How did they react?
8. How long from the time of testing did you share that you were HIV positive?

***Experience with ART***

1. When did you start taking ARVs
2. What made you take ARVs? (probe whether voluntary, sick, pressured by friends, etc)
3. What did the health provider tell you about starting ART?
4. What was your understanding about the purpose of starting ART treatment soon after being diagnosed?
5. What motivated you to start ART (probe for multiple reasons)
6. Were there any obstacles in starting ART? (Probe for support systems, did they overcome obstacle and how?)
7. Were there any obstacles in returning to the facility for follow up visits after you started ART? (Probe for reasons, did they overcome obstacle and how?)
8. Have you ever not taken your drugs when you were supposed to (probe for main reasons)
9. Did you experience any side effects of taking ARVs?
10. Did you experience any health benefits after taking ARVs?
11. Did you have anyone who regularly reminds you to take your ART? How are you related to this person?
12. Did you ever stop taking your ART for 48 hours or longer? (probe for reasons why?)

***Experience with Early Treatment Initiation***

1. Do you remember what the counselor told you about Early Treatment Initiation?
2. What did you see as the benefits of starting ART early?
3. How did you understand the purpose of the viral load test? Did a health provider discuss results from viral load tests with you? (probe for understanding of the results)

***Reasons for dropping out of HIV care (MAIN QUESTION OF THE STUDY)***

1. What made you stop ART (probe for main reasons?)
