# Supplementary material for: ‘It is not fashionable to suffer nowadays’: Community motivations to repeatedly participate in outreach HIV testing indicate UHC potential in Tanzania
Source: PLoS One. 2021 Dec 22;16(12):e0261408. doi: 10.1371/journal.pone.0261408 (PMC8694479; doi:10.1371/journal.pone.0261408)
Supplement: S1 File — (ZIP) [file pone.0261408.s001.zip › Tools/FINAL_OBSERVATION_REPORTING FORMAT.docx]

**Date:……………………….. Researcher:……………………….. Place:………………Hub:……………**

Information for researchers

Write a narrative at the end of each day (see below for example) in which you describe what you **observed a**nd **overheard** at the testing campaign. Use as much detail as possible. One of you should focus on the gazebo at three different moments in time (at the start, at the peak/dark, towards the end). The other person can also observe at different places (f.e. in the testing team car (dynamics within the team), during the show (do team-members ‘recruit and if so how’, etc.)

The goal of observing is to 1) discover routine ways of doing things and 2) to match what people say with what they do. Always pay attention to what you think is striking or different too. In your description of events you always look at three aspects: the place, the people and the activities. You can make notes on this form (but try to be as inconspicuous as possible). Always mark beginning and end-times.

An observation can also include informal conversations that you overhear. Make note in as much detail as possible, see below for an example.

The four points below are to keep you focused during your observations. You can jot down notes and then write them out in detail at night.

1. **Structural and organizational features** (describe the venue, the placing of the testing-gazebo, shows, people flow towards the gazebo, in an out of the gazebo, what is done with positive and negative tests, posters, messaging, loudspeakers, administration etc.)
2. **The activities you see** (chronologically. i.e what happens during a test: how long inside, what does the testing team do during the day, i.e how do they ‘recruit’, wat are odd things, what is happening outside of gazebo, in the main event, is there always a specific sequence of activities at the gazebo, what do people do in the queue?).
3. **People** (number of both testers/provider – male/female ratio, how they behave in the queue, after the event, interact, dress, client-provider interaction (what are powerrelations that you can observe, shouting, empathy, soft-spoken, drunk), do testers/counselors behave differently towards young/male/female/town/village, what do they say in car), are people accompanied or not? Note conversations on testing, on HIV prevention, on ART, comments on messaging, conversations between clients and testers and anything you find noteworthy etc (see below)

1. **Personal/reflective notes:** (Include attitude health providers/clients to you: refusal rates interviews/observations + reason)

| **Date:…………………….** | **Researcher:…………………... Testing event:……………………….** | **Yes/No** | **Comments** |
| --- | --- | --- | --- |
| **Physical space/confidentiality** | Gazebo available |  |  |
|  | No. of Gazebo’s |  |  |
| **Clientele** | How many clients test for HTC/day, mostly wo(men)/young/old/couple |  |  |
|  | Total People attended |  |  |
|  | Total nr of People HIV+ Include newly tested/re-test |  |  |
| **Staff** | How many staff/roles (counselor, CHV etc) |  |  |
|  | Recruitment for testing actively done during event? |  |  |
| **Pre-test Counseling** | Pre-test counselling done individually? or in group |  |  |
| *(note down total time)* | Basic facts on HIV |  |  |
|  | Transmission (including PMTCT/Couple testing) |  |  |
|  | Possible test results |  |  |
|  | Testing procedure |  |  |
|  | Consent: explained choice to agree/refuse |  |  |
|  | Any information on early treatment initiation. Note what was said? |  |  |

**Checklist after event**

**Example observation**

**22.04.2009**

Arrived at the CCC at 8.20am; the waiting bay looks full though two benches at the back are not occupied. There are 13 patients, 10 women and three men. One mother is with a girl aged about 10 years old. The three men aged above 50 years are sitting on the front bench. All patients are casually dressed.

The health provider begins to talk in mother tongue and one of the men asks her to talk in Kiswahili because he doesn’t understand Kikuyu. (I was happy at the request). Unfortunately the health provider never gave the health talk but instead she introduced us. (Just don’t know why she ended up not giving the talk after she was asked to speak in Swahili by a patient). Weighing starts and we go to our work station where patients who are voluntarily willing to participate in the discussion come to us.

10.00am while outside I overheard the nurse shouting in her mother tongue about the CD4. That some patients’ CD4 is going down while it should be going up when on drugs. All patients are very quiet.

**Informal talk with a non-user who had brought a very sick spouse to the CCC**

At 10.30 am, a man is wheeled to CCC on a wheel chair. He is accompanied by two men who are dressed casually and a lady who is casually dressed with neatly plaited hair. When they reach at the reception the two men and lady accompanying the man come out of the CCC and sit on the bench next to my work station. Once I am done with one of my interviewee and waiting for the next, the lady who accompanied the man on the wheel chair approaches me. She says she is the wife to the man on the wheel chair. Her first question to me was that “Do people who are not sick become sick when they are put on ARVs?” I reply “ARVS work differently for different people…..” She says her husband is HIV positive with a CD4 of 17. He has been very sick that he cannot walk but she has never been sick herself. She asks if she could be infected with HIV though she has never been sick and if someone who is not sick can be put on ARVs. I told her to be put on ARVs will depend on the CD4 count and the viral load. Then she asks “For how long one is supposed to take ARVs?” I reply “ARVs are for life”. I ask her if she knows her HIV status and she says she has not tested and she is not going to test for HIV for now because her priority is to take care of her husband now and after the husband improves that is when she will consider taking HIV test. She asks, “Could be infected yet I never been sick?” I ask her if she had unprotected sex with her husband before and she says yes but when the husband was too sick they did not have sex. I tell her she could be or could not be infected but the only way she can know is to go for HIV test. She became more interested in knowing how one cannot be infected when they had sexual contact with HIV infected person. Thinking out loud she said, “I wish I am not infected, I can be very happy”. She then asks me if her children could be infected. She has two children; the first is 6 and half years and the second is 4 year old. I tell her to test herself so that she can know if her children are infected or not, then they can be started on septrine or ARVs. Then she says “I am very worried for the children”. Then she asks the use of septrine and if septrine prevents one from getting infected with HIV (maybe she was thinking about taking self prescribed septrine to prevent her from infection)

Then she asks, “Where do I go for HIV testing?” I tell her behind the CCC there is a VCT, she can go there for counseling and take the test or she can go for counseling with one of the nurses are the CCC then make up her mind about the testing later. She then tells me she doesn’t want to go to the VCT now.

The nurse in charge shows up and I ask the lady to talk to the nurse. She approaches the nurse and they talk as they walk into the CCC. After about 3 minutes the lady comes out of the CCC and goes straight to the husband who was waiting outside.

**Date: 23/04/2009**

The main provincial hospital in Central province, offers both in patients and out patient services. The OPD has several departments and is always packed, most by men and women aged above 25-60 years with majority in age bracket 30-55 years. The OPD has several sections including MCH, general conditions, youth clinic, emergency, minor theater, records, pharmacy, ENT, Chest clinic, dental clinic, Eye clinic, Physiotherapy, laboratory, VCT and CCC clinic. The in patient section includes maternity, male and female medical wards, surgical wards and main theater.

The facility runs 24 hours though most outpatient departments, except general outpatient clinic, operate from 8.00-5.00pm. The building is old but every morning cleaners washing the place. All OPD sections are packed, very packed in morning hours. At the main OPD where general conditions are seen, most patients are in age range of 30-60. Females are more than men especially at the MCH where I only saw one man among 15 women at 2.00pm. Some patients are accompanied and as their patients line for consultations, those who accompany them sit out on the pavement adjustment to the consultation rooms. Here there are 5 consultation rooms. Patients are packed both in the morning and in the afternoons. The youth clinic is relatively small and has a television. The waiting bay can accommodate 10 patients at ago. The male patients come an accompanied but the female are accompanied by female companions. On Wednesday 23/04/2007 11.am I see two young men and one female at youth waiting bay. At 2.00pm I saw two young female of age range 14-16 seated next to two adults females aged above 45 years old.

In the general conditions, MCH and youth clinic HIV testing and counseling is done in the consultation rooms. Each consultation room is used by two health providers, when TC is being done one of the HP leaves the room to allow HTC exercise to take place, while other patients waiting are put on hold. The emergency and minor theater has less activity but most patronized by adult patients aged above 40 years old. Majority are female patients.

ENT, chest and physiotherapy are in one building. While ENT is very packed both morning and afternoon, the chest clinic is packed in the morning hours. I observed this during our introduction by the OPD matron in the afternoon, when I went for KII appointments in the afternoon and to conduct the interviews in the afternoon. The interviews were from 3.00pm to 5.00. At this time the chest clinic only had two patients but the ENT was packed as I was living at 5.00pm. Majority of the patients are adults, we also have some children. ENT and chest clinic are in same room but go to different consultation rooms. In ENT DTC is practiced while chest all patients are offered PITC.

In chest clinic HIV testing is done by the nurse or the clinical officer in their offices. In the chest clinic the clinical officer room is the consultation room while the nurse station is where the drugs, records and all files kept and HIV testing done. The nurse station is very spacious room.

In the ENT the HIV testing is done in the clinician and nurse office. The clinician and the nurse share a small office. In this clinic there is a records room where all patients’ records are kept. The consultant room is adjacent to the CO and nurse office. HIV counseling and testing is done in CO/nurse office. When I was there the cleaner kept coming in.

Between general OPD, ENT/chest, Pharmacy, eye clinics is the VCT and CCC. The VCT faces the general OPD washrooms but has less activity. This is the only place with VCT or HTC sign. In all departments I have not seen any IEC materials for HIV/AIDS, not even posters. There is a small poster on all consultation rooms supported by Safaricom on breast cancer which says Lets get tested now. Other places there are posters on malezi bora, not on HTC or HIV/AIDS in general. His is even in medical wards where I visited. The only place where HIV is mention is on board showing charges of services at the OPD entrance indicting among other services HIV test is free but does not indicate where one can ask for HIV test.

Apart from the VCT and PITC there is a specific HTC counseling room for patients who don’t want to go to VCT. Patients who go through this counseling are sent to laboratory for HIV testing with a referral form from the counselor and the test results are given to the counselor who then discloses the results to the patient.
