# Supplementary material for: ‘It is not fashionable to suffer nowadays’: Community motivations to repeatedly participate in outreach HIV testing indicate UHC potential in Tanzania
Source: PLoS One. 2021 Dec 22;16(12):e0261408. doi: 10.1371/journal.pone.0261408 (PMC8694479; doi:10.1371/journal.pone.0261408)
Supplement: S1 File — (ZIP) [file pone.0261408.s001.zip › Tools/FINAL_ClientsNotLinked_EN.doc]

**Clients Not Linked**

1. Can you tell me about the day you tested, what convinced you to go to get tested? (also confirm if the campaign was used to confirm)
2. What did the counselor tell you during testing ? (probe about life, staying healthy, starting treatment)
3. How did you receive the news that you were HIV positive
4. Did you any space to ask questions and were your questions answered?
5. Did you ever test anywhere else before
6. Did you go somewhere to confirm results afterwards
7. Was there anybody that helped you make that decision
8. At first when you receive the news that you are positive, did you have thoughts of committing suicide

**Not linking**

9. We know that you have not yet started treatment. What made decide **NOT TO** start treatment
10. What do you know about the benefits of starting early . *If no, briefly explain: health benefits and preventive tool*
11. Do you know about treatment as prevention
12. We completely respect your decision but for us it is good to know what makes people use treatment and what not.
13. Is there any one who influenced your decision?
14. How do you see the CHW/HBC in your community (trust, confidentiality)
15. What do you think of the Health facility/HUB (trust, confidentiality)
16. Was there someone like a traditional healer/religious leader that adviced you? What?
17. Do you use any services to keep your body healthy
    a. Lifestyle (nutrition, exercise, sex life)
    b. Drugstores (septrin other)
    c. Traditional herbs
    d. Attending prayers
18. We know sometimes people share medicines they get from the clinic. What can you say about medicines sharing in your family. *Then narrow down to ART*
19. How did the news of being HIV positive change your life.
    a. Sexual Relationships
    b. Work
    c. Children/dependents
    d. Education
20. Did you talk to anyone about being positive just after testing? Who and why this person?
21. And right now, have you talked about your status? If no what makes you not to disclose (Go to q 33)
22. How did you go about disclosing your status?
23. How did people react? What did you expect would be their reaction?
24. How do you feel the community sees you?
25. Do you think your neighbours talk about your health condition?
26. When you are in public and anyone talks about HIV, how do you feel when people are talking about HIV
27. Do they ever talk personally about HIV to you and if they talk what do they talk
28. Have you had any assistance from a traditional healer? For example for dealing with concerns you have about having this condition?
29. What is it that has changed in your lifeplans now that you are diagnosed. Probe (cutting of plans, having family
30. Would there be any time that you feel you would like to start treatment?
31. Now we discussed all these things, what have you taken from that?
