# Supplementary material for: ‘It is not fashionable to suffer nowadays’: Community motivations to repeatedly participate in outreach HIV testing indicate UHC potential in Tanzania
Source: PLoS One. 2021 Dec 22;16(12):e0261408. doi: 10.1371/journal.pone.0261408 (PMC8694479; doi:10.1371/journal.pone.0261408)
Supplement: S1 File — (ZIP) [file pone.0261408.s001.zip › Tools/FINAL_IDI Refusers_En.doc]

**SOCIAL SCIENCE TOOLS**

**IDI**

**Refusers**

1. Sex, Age, Marital Status, Location, Occupation, Education
2. Have you ever heard of HIV-testing campaigns in this community?
3. What do you think of the campaigns?
4. Have you ever been to one? If yes, did you test? [***If the person says ‘yes’ go to retesters IDI***]
5. Have you ever been to another place for HIV testing? Or use a self-test [***If the person says ‘yes’ go to retesters IDI***]

We came to you today because we know that **not** everyone wants to go for HIV-testing at these campaigns or even **do not** want to go for testing at all. We are fine with that, we did not come to this community to convince people to come for HIV-testing but to hear their opinion.

6. So you said that you have never tested for HIV? Please tell me what prevented you from going for testing?
7. What do you think will happen to you when you are found HIV positive (death, pressure)
8. Have you ever seen someone who is HIV positive .
9. If yes, what did you think of what happened to that person?
10. Have you ever heard of any support that people who are diagnosed HIV positive receive (counseling, treatment)
11. If yes, what do you know about support
12. Have you ever heard about testing early and starting treatment when you are still healthy
13. Do you ever discuss testing for HIV with your friends?
14. What do you tell eachother?
15. Is there anything in your life that make you think you are at risk for HIV?
16. Apart from HIV testing, would you prefer any other medical check-ups
17. Have you done any other tests in the past?
18. Why is it important for you to do other health tests?
19. What did you hear about the testing in the media
20. If you see the testing teams around, and the tents, do you feel in any way that you need to test?
