# Supplementary material for: ‘It is not fashionable to suffer nowadays’: Community motivations to repeatedly participate in outreach HIV testing indicate UHC potential in Tanzania
Source: PLoS One. 2021 Dec 22;16(12):e0261408. doi: 10.1371/journal.pone.0261408 (PMC8694479; doi:10.1371/journal.pone.0261408)
Supplement: S1 File — (ZIP) [file pone.0261408.s001.zip › Tools/FINAL_IDI Guide re-testers_EN.doc]

**Testing experience**

1. We met at a campaign. What did you think about this campaign?
2. Have you ever been to another place for HIV testing? Or use a self-test [*If the person says ‘yes’ go t oretesters IDI*]
3. Can you tell me about the day you tested, what convinced you to go to get tested?
4. How did the counselor do the test? (probe for the testing practice)
5. How did you feel about this?
6. Was there anybody that helped you make that decision.

*If no info from Q2: In the recruitment interview you said you wanted to test to come because…….*

7. What did the counselor tell you? (probe about life, staying healthy, starting treatment)
8. Did you any space to ask questions and were your questions answered?

**Testing under T&T**

9. What messages have you heard about testing (from the government, on radio, tv)
10. What do you think about these messages
11. What do you know about Test And Treat? (probe for health and prevention)
12. If you compare to the past, before the Test and Treat started, what are differences in how people go for testing?
13. Have your ideas about testing changed compared to before?
14. Do you feel sometimes as if you testing is a must?
15. What makes you feel that you must test?
16. Who do you think is providing the testing services?
17. What do you feel about these services being provided nearby (by that provider,probe for if people feel obliged)
18. Does the setting of the services matter to how you trust/experience the testing? (probe)
19. What do you think will happen to you when you are found HIV positive (death, pressure)
20. Have you ever seen someone who is HIV positive .
21. Have you ever heard of any support that people who are diagnosed HIV positive receive (counseling, treatment)
22. If yes, what do you know about support
23. Have you ever heard about testing early and starting treatment when you are still healthy
24. Do you ever discuss testing for HIV with your friends?
25. What do you tell eachother?
26. Is there anything in your life that make you think you are at risk for HIV?
27. Apart from HIV testing, would you prefer any other medical check-ups

**Only for re-testers**

28. We know you tested several times in your life. You said you tested because…..insert from the recruitment. Can you explain more about this (if people doubt, is it becauase of feeling at risk, or because of not trusting the device?)
29. YOu were given a card at the Campaign with a date to re-test, do you normally observe that date?
30. How does testing assist you in observing your health? (ask for risk behavior/condom use?)
31. Do you normally trust the results? If yes, what makes you trust, if No, what makes you not trust
32. Tell me about teh situation where you trusted the test and a situation where you had doubts.
33. From the way the test was done the day we recruited you, did you trust the results?
34. Have you gone to test somewhere else since?
35. We know you tested in different facilities. Can you tell us differences in the way they use the rapid tests?
36. Do you have concerns about the services, counseling,
