# Supplementary material for: ‘It is not fashionable to suffer nowadays’: Community motivations to repeatedly participate in outreach HIV testing indicate UHC potential in Tanzania
Source: PLoS One. 2021 Dec 22;16(12):e0261408. doi: 10.1371/journal.pone.0261408 (PMC8694479; doi:10.1371/journal.pone.0261408)
Supplement: S1 File — (ZIP) [file pone.0261408.s001.zip › Tools/FINAL_IDI Guide re-testers_Swah.doc]

**Uzoefu wako wakati wa kupima**

1. Tulikutana katika kampeni ya upimaji, ulionaje kampeni ile?
2. Umewahi kwenda mahali pengine kwa ajili ya kupima VVU? Au umewahi kutumia kipimo cha kujipima VVU mwenyewe? [*Ikiwa atasema ‘ndiyo’ nenda dodoso/kipengele cha wanaorudia kupima*]
3. Unaweza kunielezea kuhusiana na siku ulipopima? Ni nini kilikushawishi kwenda kupima?
4. Mshauri nasaha alifanyaje kipimo? (Dodosa jinsi kipimo kilivyofanyika)
5. Ulijisikiaje kwa jinsi kipimo kilivyofanyika?
6. Ni nani alikusaidia kufanya maamuzi ya kwenda kupima?

*Ikiwa hana la kusema: wakati ulipohojiwa kuandikishwa ulisema ulipenda kuja kupima kwa sababu ..........*

7. Mshauri nasaha alikuambia nini? (dodosa kuhusiana na maisha, kuendelea kuwa na afya, kuanza kutumia tiba)
8. Ulipata nafasi yoyote kuuliza maswali? Ikiwa ndiyo, maswali yako yalijibiwa?
9. Ikiwa ndiyo, uliuliza nini na ulijibiwaje?

**Kupima kupitia mradi wa UPIMAJI na TIBA**

10. Kuna jumbe gani umezisikia kuhusiana na upimaji? (kutoka serikalini, kwenye radio, television)
11. Una mawazo gani juu ya jumbe hizi?
12. Unafahamu nini kuhusiana na UPIMAJI na TIBA? (Dodosa kuhusu afya na uzuiaji wa VVU)
13. Ukilinganisha na zamani, kabla sera ya UPIMAJI na TIBA kuanza, kuna tofauti gani kwenye namna watu wanavyokwenda kupima?
14. Je, mawazo yako juu ya upimaji yamebadilika ukilinganisha na kabla ya sera?
15. **Ukijichunguza ndani yako;** Je, kuna wakati unajisikia kama kupima ni lazima?
16. Ni nini **kinaweza** kukufanya ukajisikia kwamba kupima ni lazima?
17. Unadhani ni nani wanaotoa hizi huduma za upimaji?
18. Unajisikiaje unapoona hizi huduma zinatolewa maeneo ya karibu [kutoka kwa huyo anayetoa] (**Dodosa**: kufahamu ikiwa watu wanajisikia kuwiwa kupima)
19. Je, inajalisha mahali huduma za upimaji zinapotolewa ikilinganishwa na unavyoamimi au kupokea upimaji? (Dodosa)
20. Unadhani ni nini kitatokea kwako ikiwa utagundulika una maambukizi ya VVU? (Dodosa: kifo au mshtuko?)
21. Umewahi kumuona mtu ambaye ana maambukizi ya VVU?
22. Umewahi kusikia kuhusiana na msaada ambao watu waliopimwa na kukutwa na VVU wanapokea? (ushauri, tiba)
23. Ikiwa ndio, unafahamu nini kuhusiana na msaada huo?
24. Umewahi kusikia kuhusiana na kupima mapema na kuanza tiba ukiwa una afya nzuri?
25. Huwa mnajadili nini na rafiki zako kuhusiana na upimaji?
26. Huwa mnaambiana nini?
27. Katika maisha yako ni nini kinakufanya ufikirie upo hatarini kuambukizwa VVU?
28. Ukiachana na kupima VVU, unapendelea kupima vipimo gani vingine vya afya?

**Kwa wale tu wanaorudia-rudia kupima**

29. Tunafahamu kwamba umewahi kupima VVU mara kadhaa maishani mwako. Ulisema ulipima kwa sababu ..... [*sema alichosema wakati wa kuandikishwa*]. Unaweza kuelezea zaidi? [watu wakiwa wana mashaka, ni kwa sababu wanajihisi wapo hatarini au kwa sababu ya kutokuamini kipimo?]
30. Ulipewa kadi wakati wa kampeni ya upimaji ikiwa na tarehe ya kurudia kupima, je kwa kawaida unazingatia tarehe hiyo?
31. Ni kwa namna gani kupima inakusaidia kufuatilia afya yako? (Dodosa: tabia hatarishi/matumizi ya kondomu)
32. Kwa kawaida, huwa unaamini majibu ya kipimo? Ikiwa ndiyo, ni nini kinakufanya uamini? Ikiwa hapana, ni nini kinakufanya usiamini?
33. Hebu nielezee kwa wakati uliamini kipimo, na pia nielezee kwa wakati ulipokuwa na mashaka na kipimo.
34. Kwa kuangalia siku ulipopimwa siku ulipoandikishwa, uliamini majibu ya kipimo?
35. Baada ya siku ile, ulienda kupima wapi tena?
36. Tunafahamu umewahi kupima sehemu tofauti-tofauti, tunaomba utuelezee utofauti ni wa namna gani kipimo cha haraka kinavyofanyika.
37. Tuelezee kuhusiana na hoja nyingine zozote ulizo nazo kuhusiana na huduma za upimaji na ushauri nasaha.
