# Supplementary material for: ‘It is not fashionable to suffer nowadays’: Community motivations to repeatedly participate in outreach HIV testing indicate UHC potential in Tanzania
Source: PLoS One. 2021 Dec 22;16(12):e0261408. doi: 10.1371/journal.pone.0261408 (PMC8694479; doi:10.1371/journal.pone.0261408)
Supplement: S1 File — (ZIP) [file pone.0261408.s001.zip › Tools/FINAL_ClientsLinked_EngSwah.doc]

- ***People that delayed linking but are in care***
- ***People that linked and they are in care for at least one month***
- ***People that have tested while being positive in care***

1. Can you tell me about the day you tested, what convinced you to go to get tested?
2. Hebu nielezee jinsi ilivyokua ile siku ulipopima kwenye kampeni. Ni nini kilikushawishi mpaka ukaenda kupata huduma ya kupima?
3. Was there anybody that helped you make that decision.

*If no info from Q2: In the recruitment interview you said you wanted to test to come because…….*

4. Ni nani alikusaidia kufanya maamuzi ya kwenda kupima?

*If no info from Q2: In the recruitment interview you said you wanted to test to come because…….*

*Kama hakuna maelezo kwa swali la pili: Wakati wa kuandikishwa ulisema ulipenda kupima kwa sababu .............*

5. What did the counselor tell you? (probe about life, staying healthy, starting treatment)
6. Yule mshauri nasaha alikuambia nini? (**Dodosa***: kuhusiana na maisha, kuendelea kuwa na afya, kuanza kutumia tiba*)
7. How did you receive the news that you were positive
8. Ulipokeaje taarifa kwamba umeambukizwa VVU?
9. Did you have any space to ask questions and were your questions answered?
10. Ulipata nafasi yoyote kuuliza maswali? Ikiwa ndiyo, maswali yako yalijibiwa?
11. Ikiwa ndiyo, uliuliza nini na ulijibiwaje?
12. Can you tell me how you started treatment/ or why you decided not to start treatment?
13. Tafadhali hebu niambie, ulianzaje-anzaje kutumia dawa za ART? Au ni kwa nini uliamua kutokuanza kutumia dawa za ART?
14. **For those on ART***: Why did you go to test again?*
15. **Kwa wale wanaotumia ART***: Ni kwa nini ulienda kupima tena?*

**Delay clinic / Kuchelewa kliniki/ kujiunga na huduma**

1. How did you go to the health facility for the first time?
2. Hebu nielezee, ulifikaje katika kituo ch afya ile mara ya kwanza?

***Probe****: support from HBC/CHW/or alone/friend/TH/RelL/Community person and how did you experience this support?*

*if immediately go to Q12, if there was a delay continue*

***Dodosa****: Vipi ulipata msaada kutoka kwa mhudumu wa afya ngazi ya jamii au ulienda peke yako au na rafiki au na mganga wa jadi au na kiongozi wa kidini au mwanajamii tu? Hebu tuelezee uzoefu wako juu ya msaada huu?*

3. Why did you not go immediately?
4. Ni kwa nini haukwenda kituo cha afya moja-kwa-moja?
5. Did you go anywhere else to confirm the results?
6. Ulikwenda mahali gani pengine kuthibitisha/ kuhakiki majibu ya kipimo?
7. Did/do you use traditional healers, medicines from the drug store, prayers, herbs?
8. Ulitumia tiba gani nyingine? (**Dodosa**: *Tiba za jadi, dawa kutoka duka la dawa, kuombewa, mitishamba etc.*)
9. We know sometimes people share medicines they get from the clinic. What can you say about medicines sharing in your family. *Then narrow down to ART*
10. Tunaelewa kwamba kuna wakati watu wanashirikiana dawa wanazopewa hosipitali. Unaweza ukaliongeleaje suala hili la kushirikiana dawa katika kaya yako? (*Kisha, endelea kuuliza kuhusiana na kushirikiana dawa za ART*)

**First time clinic/ Siku ya kwanza kufika kliniki**

11. Tell me about your experience at the health facility on that day. What were the steps
12. Hebu nielezee kuhusiana na uzoefu wako ulipokuwa kituo cha huduma za afya ile siku. Ni hatua gani ulizipitia katika kupata huduma?
13. Did you get additional counseling? (did you speak to an expert client?) If yes, what did they tell you?
14. Ulipata ushauri nasaha wa ziada? (Ulipata nafasi ya kuongea na mtu aliyebobea kutumia dawa za ART?) Ikiwa ndiyo, alikuambia nini?
15. How did you feel being in the CTC for the first time?
16. Ulijisikiaje kuwepo katika kituo cha huduma na tiba kwa mara ya kwanza?
17. Were you told to attend a class? If no go to 18?
18. Uliambiwa uhudhurie darasa? (*Ikiwa hapana nenda swali la 18*)
19. What were the main things you learned?
20. Ni vitu gani vya msingi sana ulivyojifunza?
21. What were you told about tests you will receive
22. Uliambiwa nini kuhusiana na vipimo ambavyo utapewa?

**Probe**: difference CD4/VL (kiwango cha Kinga)

**Dodosa**: Tofauti kati ya kiwango cha kinga na wingi wa virusi

What do you understand these test to be?

Unaelewa ni nini vipimo hivi vinahusisha?

23. Have you thought about going somewhere else to another facility? And if so why?
24. Umewahi kufikiria kuhusiana na kwenda mahali pengine kwenye kituo kingine cha huduma za afya? Ikiwa ndivyo, ni kwa nini?
25. How do you feel about the current way of reminders from the health facility? What would you prefer? (probe for text messages)
26. Unajisikiaje kuhusiana na namna ya sasa hivi ya kukumbushwa kwenda kituo cha huduma na tiba inayotumika pale kituo cha afya? Wewe ungependelea nini? (**Dodosa**: Kutumiwa ujumbe mfupi wa maneno)

**Treatment/ Tiba**

We want to ask you now about your experiences with treatment

Tunapenda kukuuliza sasa kuhusiana na uzoefu wako katika kutumia tiba

27. Did you have any side effects
28. Ulipata madhara yoyote yatokanayo na tiba?
29. How do you take your drugs?
30. Unatumiaje/ unamezaje dawa zako?
31. Are there maybe some days that you do not take your pills? Reasons
32. Hebu nielezee kuhusiana na siku ambazo labda huwa haumezi vidonge vyako. Sababu huwa ni nini?
33. How do you make sure you remember to take your pills? Probe, somebody, technique, changes …
34. Ni kwa namna gani huwa unahakikisha unakumbuka kumeza vidonge vyako? (**Dodosa**: Una mtu, mbinu, mabadiliko....)
35. How do you feel about the distance to the health facility (probe for shifting between HF)
36. Unajisikiaje kuhusiana na umbali kilipo kituo cha afya? (**Dodosa**: kuhama kituo)
37. Apart from the service you are receiving at the facility, how do you take care of yourself and your body? Probe for eating, alcohol, sex, exercise, pacing work.
38. Ukiachana na huduma unazopata kituo cha afya, ni kwa namna gani unajihudumia wewe na mwili wako? (**Dodosa**: Unavyokula, pombe, ngono, mazoezi, mpangilio wako wa kazi...)
39. Have you ever thought about not continuing treatment
40. Hebu nielezee kuhusiana na wakati wowote ulipowaza kuhusiana na kuacha kutumia dawa?
41. Does being on life-time treatment make you maybe emotional or worry?
42. Je, kutumia tiba kwa maisha yako yote inakufanya unakua mnyonge au na wasiwasi? Hebu nielezee mtazamo wako juu ya tiba hii ya maisha yako yote.
43. Are there places where you were recently (hospital, workplace, school, harusi, dance) where you did not want to take your medicines/reveal
44. Ni maeneo gani huwa unakuwepo ambapo haupendi kuonekana wakati unatumia dawa zako? (**Dodosa**: Hospitali, kazini, shule, harusini, ngoma, michezo, ibadani....)
45. **A: For non-testers**: Have you ever thought about testing again, what would be reasons for you to test again, while on treatment?
46. **A: Kwa wasiopima:** Umewahi kuwaza kuhusu kupima VVU tena? Ni nini inaweza kuwa sababu ya wewe kwenda kupima tena wakati unatumia tiba?

**B: For testers**: we know you tested at the campaign whilst on treatment and we would like to ask why?

B: **Kwa wanaopima**: Tunafahamu kwamba ulipima kwenye kampeni wakati unatumia tiba, tungependa kufamu ni nini sababu za wewe kwenda kupima?

**HIV and life / VVU na maisha**

47. How did the news of being HIV positive change your life.
    a. Sexual Relationships
    b. Work
    c. Children/dependents
48. Ni kwa namna gani taarifa ya kwamba una maambukizi ya VVU imebadili maisha yako?
    a. Mahusiano yako ya kimapenzi
    b. Kazi
    c. Watoto/wanaokutegemea
49. Did you talk to anyone about being positive just after testing? Who and why this person?
50. Ni nani uliongea naye kuhusu wewe kuwa na maambukizi ya VVU mara tu baada ya kupima? Kwa nini uliongea naye? (If s/he talked to somebody then go to qn ..
51. And right now, have you talked about your status? If no what makes you not to disclose (Go to q 33)
52. Mpaka sasa, umeongea na mtu yeyote kuhusu hali yako ya maambukizi ya VVU? Ikiwa hapana, ni nini kinakufanya usiweke wazi hali yako ya maambukizi? (??Go to qn ...)
53. How did you go about disclosing your status?
54. Ulianzaje kuongea kuweka wazi kuhusu hali yako ya maambukizi ya VVU?
55. How did people react? Did you expect that reaction?
56. Watu walipokeaje hali hiyo? Ulitarajia watu wangepokeaje hali hiyo?
57. How do you feel the community sees you?
58. Ukijichunguza, unajisikia jamii inakuonaje wewe?
59. Do you think your neighbours talk about your health condition?
60. Majirani zako wanaongeleaje hali yako ya kiafya?
61. Have you had any assistance from a traditional healer? For example for dealing with concerns you have about having this condition?
62. Umewahi kupata msaada gani kutoka kwa mganga wa kienyeji? Kwa mfano, namna ya kukabiliana na hoja ulizo nazo kuhusiana na hali hii?
63. What is it that has changed in your lifeplans now that you are diagnosed. Probe (cutting of plans, having family
64. Ni kitu gani ambacho kimebadilika katika mipangilio yako ya kimaisha kwa sasa baada ya kugundulika una VVU? (**Dodosa**: Kusitisha mipango, kuwa na familia..)
