# Supplementary material for: ‘It is not fashionable to suffer nowadays’: Community motivations to repeatedly participate in outreach HIV testing indicate UHC potential in Tanzania
Source: PLoS One. 2021 Dec 22;16(12):e0261408. doi: 10.1371/journal.pone.0261408 (PMC8694479; doi:10.1371/journal.pone.0261408)
Supplement: S1 File — (ZIP) [file pone.0261408.s001.zip › Tools/FINAL_ClientsLinked__Swah.doc]

- ***People that delayed linking but are in care***
- ***People that linked and they are in care for at least one month***
- ***People that have tested while being positive in care***

1. Hebu nielezee jinsi ilivyokua ile siku ulipopima kwenye kampeni. Ni nini kilikushawishi mpaka ukaenda kupata huduma ya kupima?
2. Ni nani alikusaidia kufanya maamuzi ya kwenda kupima?

*Kama hakuna maelezo kwa swali la pili: Wakati wa kuandikishwa ulisema ulipenda kupima kwa sababu .............*

3. Yule mshauri nasaha alikuambia nini? (**Dodosa***: kuhusiana na maisha, kuendelea kuwa na afya, kuanza kutumia tiba*)
4. Ulipokeaje taarifa kwamba umeambukizwa VVU?
5. Ulipata nafasi yoyote kuuliza maswali? Ikiwa ndiyo, maswali yako yalijibiwa?
6. Ikiwa ndiyo, uliuliza nini na ulijibiwaje?
7. Tafadhali hebu niambie, ulianzaje-anzaje kutumia dawa za ART? Au ni kwa nini uliamua kutokuanza kutumia dawa za ART?
8. **Kwa wale wanaotumia ART***: Ni kwa nini ulienda kupima tena?*

**DELAY CLINIC / KUCHELEWA KLINIKI/ KUJIUNGA NA HUDUMA**

1. Hebu nielezee, ulifikaje katika kituo ch afya ile mara ya kwanza?

*if immediately go to Q12, if there was a delay continue*

***Dodosa****: Vipi ulipata msaada kutoka kwa mhudumu wa afya ngazi ya jamii au ulienda peke yako au na rafiki au na mganga wa jadi au na kiongozi wa kidini au mwanajamii tu? Hebu tuelezee uzoefu wako juu ya msaada huu?*

2. Ni kwa nini haukwenda kituo cha afya moja-kwa-moja?
3. Ulikwenda mahali gani pengine kuthibitisha/ kuhakiki majibu ya kipimo?
4. Ulitumia tiba gani nyingine? (**Dodosa**: *Tiba za jadi, dawa kutoka duka la dawa, kuombewa, mitishamba etc.*)
5. Tunaelewa kwamba kuna wakati watu wanashirikiana dawa wanazopewa hosipitali. Unaweza ukaliongeleaje suala hili la kushirikiana dawa katika kaya yako? (*Kisha, endelea kuuliza kuhusiana na kushirikiana dawa za ART*)

**FIRST TIME CLINIC/ SIKU YA KWANZA KUFIKA KLINIKI**

6. Hebu nielezee kuhusiana na uzoefu wako ulipokuwa kituo cha huduma za afya ile siku. Ni hatua gani ulizipitia katika kupata huduma?
7. Ulipata ushauri nasaha wa ziada? (Ulipata nafasi ya kuongea na mtu aliyebobea kutumia dawa za ART?) Ikiwa ndiyo, alikuambia nini?
8. Ulijisikiaje kuwepo katika kituo cha huduma na tiba kwa mara ya kwanza?
9. Uliambiwa uhudhurie darasa? (*Ikiwa hapana nenda swali la 18*)
10. Ni vitu gani vya msingi sana ulivyojifunza?
11. Uliambiwa nini kuhusiana na vipimo ambavyo utapewa?

**Dodosa**: Tofauti kati ya kiwango cha kinga na wingi wa virusi

Unaelewa ni nini vipimo hivi vinahusisha?

12. Umewahi kufikiria kuhusiana na kwenda mahali pengine kwenye kituo kingine cha huduma za afya? Ikiwa ndivyo, ni kwa nini?
13. Unajisikiaje kuhusiana na namna ya sasa hivi ya kukumbushwa kwenda kituo cha huduma na tiba inayotumika pale kituo cha afya? Wewe ungependelea nini? (**Dodosa**: Kutumiwa ujumbe mfupi wa maneno)

**TREATMENT/ TIBA**

Tunapenda kukuuliza sasa kuhusiana na uzoefu wako katika kutumia tiba

14. Ulipata madhara yoyote yatokanayo na tiba?
15. Unatumiaje/ unamezaje dawa zako?
16. Hebu nielezee kuhusiana na siku ambazo labda huwa haumezi vidonge vyako. Sababu huwa ni nini?
17. Ni kwa namna gani huwa unahakikisha unakumbuka kumeza vidonge vyako? (**Dodosa**: Una mtu, mbinu, mabadiliko....)
18. Unajisikiaje kuhusiana na umbali kilipo kituo cha afya? (**Dodosa**: kuhama kituo)
19. Ukiachana na huduma unazopata kituo cha afya, ni kwa namna gani unajihudumia wewe na mwili wako? (**Dodosa**: Unavyokula, pombe, ngono, mazoezi, mpangilio wako wa kazi...)
20. Hebu nielezee kuhusiana na wakati wowote ulipowaza kuhusiana na kuacha kutumia dawa?
21. Je, kutumia tiba kwa maisha yako yote inakufanya unakua mnyonge au na wasiwasi? Hebu nielezee mtazamo wako juu ya tiba hii ya maisha yako yote.
22. Ni maeneo gani huwa unakuwepo ambapo haupendi kuonekana wakati unatumia dawa zako? (**Dodosa**: Hospitali, kazini, shule, harusini, ngoma, michezo, ibadani....)
23. **A: Kwa wasiopima:** Umewahi kuwaza kuhusu kupima VVU tena? Ni nini inaweza kuwa sababu ya wewe kwenda kupima tena wakati unatumia tiba?

B: **Kwa wanaopima**: Tunafahamu kwamba ulipima kwenye kampeni wakati unatumia tiba, tungependa kufamu ni nini sababu za wewe kwenda kupima?

**HIV AND LIFE / VVU NA MAISHA**

24. Ni kwa namna gani taarifa ya kwamba una maambukizi ya VVU imebadili maisha yako?
    a. Mahusiano yako ya kimapenzi
    b. Kazi
    c. Watoto/wanaokutegemea
25. Ni nani uliongea naye kuhusu wewe kuwa na maambukizi ya VVU mara tu baada ya kupima? Kwa nini uliongea naye? (If s/he talked to somebody then go to qn ..
26. Mpaka sasa, umeongea na mtu yeyote kuhusu hali yako ya maambukizi ya VVU? Ikiwa hapana, ni nini kinakufanya usiweke wazi hali yako ya maambukizi? (??Go to qn ...)
27. Ulianzaje kuongea kuweka wazi kuhusu hali yako ya maambukizi ya VVU?
28. Watu walipokeaje hali hiyo? Ulitarajia watu wangepokeaje hali hiyo?
29. Ukijichunguza, unajisikia jamii inakuonaje wewe?
30. Majirani zako wanaongeleaje hali yako ya kiafya?
31. Umewahi kupata msaada gani kutoka kwa mganga wa kienyeji? Kwa mfano, namna ya kukabiliana na hoja ulizo nazo kuhusiana na hali hii?
32. Ni kitu gani ambacho kimebadilika katika mipangilio yako ya kimaisha kwa sasa baada ya kugundulika una VVU? (**Dodosa**: Kusitisha mipango, kuwa na familia..)
