# Supplementary material for: ‘It is not fashionable to suffer nowadays’: Community motivations to repeatedly participate in outreach HIV testing indicate UHC potential in Tanzania
Source: PLoS One. 2021 Dec 22;16(12):e0261408. doi: 10.1371/journal.pone.0261408 (PMC8694479; doi:10.1371/journal.pone.0261408)
Supplement: S1 File — (ZIP) [file pone.0261408.s001.zip › Tools/FINAL_ClientsNotLinked_Swah.doc]

1. Hebu nielezee kuhusiana na ile siku ulipopima, ni nini kilikushawishi kwenda kupima? (*Hakiki kuona kama alipima kwenye kampeni kupata uhakika*)
2. Yule shauri nasaha alikuambia nini wakati wa kupima? (**Dodosa**: *Kuhusiana na maisha, kuendelea kuwa na afya, kuanza kutumia tiba*)
3. Ulipokeaje taarifa kwamba umeambukizwa VVU?
4. Ulipata nafasi yoyote kuuliza maswali? Ikiwa ndiyo, maswali yako yalijibiwa?
5. Ikiwa ndiyo, uliuliza nini na ulijibiwaje?
6. Ulikuwa umewahi kwenda kupima wapi pengine kabla ya siku ile?
7. Ulikwenda wapi pengine kuhakikisha majibu yako ya kipimo baada ya majibu yale?
8. Ni nani alikuwepo karibu na wewe aliyekusaidia kufanya maamuzi hayo?
9. Mara ya kwanza ulipopokea taarifa kwamba umeambukizwa VVU, ulipata mawazo gani? (Dodosa: Mawazo ya kujiua?)

**Not linking**

Tunafahamu kwamba bado haujaanza matibabu. Na tunaheshimu sana maamuzi yako lakini kwetu sisi ni vizuri tukaelewa ni nini kinawafanya watu watumie tiba na ni nini kinawafanya wasitumie tiba.

10. Tungependa kufahamu, ni nini kimekufanya umeamua kutokuanza matibabu?
11. Unafahamu nini kuhusu manufaa ya kuanza kutumia tiba mapema? *(Ikiwa hafahamu, elezea kwa kifupi manufaa ya kiafya na jinsi inasaidia kuzuia maambukizi)*
12. Ni nani aliyekushawishi au kukupa hamasa katika kufanya maamuzi?
13. Unamuonaje mhudumu wa afya ngazi ya jamii katika jamii yako? (**Dodosa**: *Masuala ya uaminifu, usiri..*)
14. Una mtazamo gani juu ya kituo cha afya cha ........ (hub) (**Dodosa**: *Masuala ya uaminifu, usiri..*)
15. Kuna mtu yeyote mashuhuri kama mganga wa kienyeji au kiongozi wa kidini aliyekupa ushauri wowote? Ikiwa ndiyo, ushauri gani?
16. Unatumia/kupata huduma gani za kuuweka mwili wako kuwa na afya?
    a. Mfumo wa maisha? (**Dodosa**: Lishe, mazoezi, maisha ya kingono
    b. Duka la dawa (**Dodosa**: Septrin, nyingine)
    c. Mitishamba
    d. Kuhudhuria maombezi/kuombewa
17. Tunaelewa kwamba kuna wakati watu wanashirikiana dawa wanazopewa hosipitali. Unaweza ukaliongeleaje suala hili la kushirikiana dawa katika kaya yako? (*Kisha, endelea kuuliza kuhusiana na kushirikiana dawa za ART*)
18. Ni kwa namna gani taarifa ya kwamba una maambukizi ya VVU imebadili maisha yako?
    a. Mahusiano yako ya kimapenzi
    b. Kazi
    c. Watoto/wanaokutegemea
    d. Elimu
19. Ni nani uliongea naye kuhusu wewe kuwa na maambukizi ya VVU mara tu baada ya kupima? Kwa nini uliongea naye? (If s/he talked to somebody then go to qn 23)
20. Ikiwa hapana, na mpaka sasa, umeongea na mtu yeyote kuhusu hali yako ya maambukizi ya VVU? Ikiwa hapana, ni nini kinakufanya usiwe wazi hali yako ya maambukizi? (??Go to q 33)
21. Ulianzaje kuongea kuweka hali yako ya maambukizi ya VVU wazi?
22. Watu walipokeaje hali hiyo? Ulitarajia watu wangepokeaje hali hiyo?
23. Ukijichunguza, unajisikia jamii ina kuonaje wewe?
24. Majirani zako wanaongeleaje hali yako ya kiafya?
25. Unapokua na watu wengine kisha mtu akaongea kitu chochote kuhusiana na HIV, unajisikiaje pale watu wanapojadili/kuongea kuhusiana na VVU?
26. Kuna wakati watu wanaongea hasa na wewe kuhusu HIV? Ikiwa wanaongea, kwa kawaida huwa wanaongelea nini?
27. Umewahi kupata msaada gani kutoka kwa mganga wa kienyeji? Kwa mfano, namna ya kukabiliana na hoja ulizo nazo kuhusiana na hali hii?
28. Ni kitu gani ambacho kimebadilika katika mipangilio yako ya kimaisha kwa sasa baada ya kugundulika una VVU? (**Dodosa**: Kusitisha mipango, kuwa na familia..)
29. Kuna wakati wowote unajiona kama utapenda kuanza kutumia tiba?
30. Sasa, baada ya kuongea vyote tulivyoongea leo, umepata/kujifunza kitu gani kipya?
