# Supplementary material for: ‘It is not fashionable to suffer nowadays’: Community motivations to repeatedly participate in outreach HIV testing indicate UHC potential in Tanzania
Source: PLoS One. 2021 Dec 22;16(12):e0261408. doi: 10.1371/journal.pone.0261408 (PMC8694479; doi:10.1371/journal.pone.0261408)
Supplement: S1 File — (ZIP) [file pone.0261408.s001.zip › Tools/FINAL_IDI_Refusers_Swah.doc]

1. Umewahi kusikia kuhusu kampeni za upimaji wa VVU katika jamii hii?
2. Una mawazo gani kuhusiana na kampeni zile?
3. Umewahi kuhudhuria kampeni moja wapo? Ikiwa ndiyo, ulipima? [***Ikiwa anasema ndiyo, nenda dodoso la wanaorudia kupima***]
4. Umewahi kuhudhuria mahali pengine kwa ajili ya kupima VVU? Au umewahi kutumia kipimo cha kujipima mwenyewe? [***Ikiwa anasema ndiyo, nenda dodoso la wanaorudia kupima***]

Tumekuja kwako leo kwa sababu tunaelewa **SIO** kila mtu anapenda kwenda kupima katika hizi kampeni za upimaji au pia wengine hawapendi kupima kabisa. Hilo halina shida kwetu, hatukuja hapa katika jamii kuwashawishi watu kujitokeza kupima ila tunapenda kusikiliza maoni yenu tu.

5. Kwa hiyo ulisema haujawahi kupima VVU? Tafadhali, hebu niambie ni nini kilikuzuia kwenda kupima VVU?
6. Unadhani nini kitatokea kwako ikiwa utagundulika umeambukizwa VVU (**Dodosa**: Kifo, mshtuko?)
7. Have you ever seen someone who is HIV positive .
8. Umewahi kumuona mtu ambaye ana maambukizi ya VVU?
9. If yes, what did you think of what happened to that person?
10. Ikiwa ndiyo, uliwaza nini kuhusiana na kilichotokea kwa mtu yule?
11. Umewahi kusikia kuhusiana na msaada ambao watu waliopimwa na kukutwa na VVU wanapokea? (ushauri, tiba)
12. Ikiwa ndio, unafahamu nini kuhusiana na msaada huo?
13. Umewahi kusikia kuhusiana na kupima mapema na kuanza tiba ukiwa una afya nzuri?
14. Huwa mnajadili nini na rafiki zako kuhusiana na upimaji?
15. Huwa mnaambiana nini?
16. Katika maisha yako ni nini kinakufanya ufikirie upo hatarini kuambukizwa VVU?
17. Ukiachana na kupima VVU, unapendelea kupima vipimo gani vingine vya afya?
18. Umewahi kupima vipimo vingine katika kipindi cha nyuma?
19. Kwa nini ni muhimu kwako kufanyiwa vipimo vingine vya afya?
20. Ulisikia nini kuhusiana na upimaji wa VVU katika vyombo vya habari?
21. Ikiwa umewaona timu ya wapimaji maeneo ya karibu na yale mahema, kuna namna yoyote unajisikia haja ya kupima?
