# Supplementary material for: ‘It is not fashionable to suffer nowadays’: Community motivations to repeatedly participate in outreach HIV testing indicate UHC potential in Tanzania
Source: PLoS One. 2021 Dec 22;16(12):e0261408. doi: 10.1371/journal.pone.0261408 (PMC8694479; doi:10.1371/journal.pone.0261408)
Supplement: S1 File — (ZIP) [file pone.0261408.s001.zip › Tools/FINAL_ClientsLinked_EN.doc]

*People that delayed linking but are in care*

*People that linked and they are in care for at least one month*

*People that have tested while being positive in care*

1. Can you tell me about the day you tested, what convinced you to go to get tested?
2. Was there anybody that helped you make that decision.

*If no info from Q2: In the recruitment interview you said you wanted to test to come because…….*

3. What did the counselor tell you? (probe about life, staying healthy, starting treatment)
4. How did you receive the news that you were positive
5. Did you any space to ask questions and were your questions answered?
6. Can you tell me how you started treatment/ or why you decided not to start treatment?
7. *For those on ART, why did you go to to test again?*

**Delay clinic**

8. How did you go to the health facility for the first time?

*Probe: support from HBC/CHW/or alone/friend/TH/RelL/Community person and how did you experience this support?*

*if immediately go to Q12, if there was a delay continue*

9. Why did you not go immediately?
10. Did you go anywhere else to confirm the results?
11. Did/do you use traditional healers, medicines from the drug store, prayers, herbs?
12. We know sometimes people share medicines they get from the clinic. What can you say about medicines sharing in your family. *Then narrow down to ART*

**First time clinic**

13. Tell me about your experience at the health facility on that day. What were the steps
14. Did you get additional counseling? (did you speak to an expert client?) If yes, what did they tell you?
15. How did you feel being in the CTC for the first time?
16. Were you told to attend a class? If no go to 18?
17. What were the main things you learned?
18. What were you told about tests you will receive

**Probe**: difference CD4/VL (kiwango cha Kinga)

What do you understand these test to be

19. Have you thought about going somewhere else to another facility? And if so why?
20. How do you feel about the current way of reminders from the health facility? What would you prefer? (probe for textmessages)

**Treatment**

We want to ask you now about your experiences with treatment

21. Did you have any side effects
22. How do you take your drugs?
23. Are there maybe some days that you do not take your pills? Reasons
24. How do you make sure you remember to take your pills? Probe, somebody, technique, changes …
25. How do you feel about the distance to the health facility (probe for shifting between HF)
26. Apart from the service you receiving at the facility, how do you take care of yourself and your body? Probe for eating, alcohol, sex, exercise, pacing work.
27. Have you ever thought about not continuing treatment
28. Does being on life-time treatment make you maybe emotional or worry?
29. Are there places where you were recently (hospital, workplace, school, harusi, dance) where you did not want to take your medicines/reveal
30. A.For non-testers: Have you ever thought about testing again, what would be reasons for you to test again, while on treatment?

B.For testers: we know you tested at the campaign whilst on treatment and we would like to ask why?

**HIV and life**

31. How did the news of being HIV positive change your life.
    a. Sexual Relationships
    b. Work
    c. Children/dependents
32. Did you talk to anyone about being positive just after testing? Who and why this person?
33. And right now, have you talked about your status? If no what makes you not to disclose (Go to q 33)
34. How did you go about disclosing your status?
35. How did people react? Did you expect that reaction?
36. How do you feel the community sees you?
37. Do you think your neighbours talk about your health condition?
38. Have you had any assistance from a traditional healer? For example for dealing with concerns you have about having this condition?
39. What is it that has changed in your lifeplans now that you are diagnosed. Probe (cutting of plans, having family
