# Supplementary material for: ‘It is not fashionable to suffer nowadays’: Community motivations to repeatedly participate in outreach HIV testing indicate UHC potential in Tanzania
Source: PLoS One. 2021 Dec 22;16(12):e0261408. doi: 10.1371/journal.pone.0261408 (PMC8694479; doi:10.1371/journal.pone.0261408)
Supplement: Attachment — Submitted filename: Response to Reviewers Comments_PLOS ONE.edited.docx [file pone.0261408.s002.docx]

Reviewer’s comments

1. The study presents the results of the original research.

Reviewer: Yes

2. Results reported have not been published elsewhere.

Reviewer: Yes

3. Experiments, statistics, and other analyses are performed to a high technical standard and are described in sufficient detail.

Reviewer: Yes

4. Conclusions are presented appropriately and are supported by the data.

Reviewer: Yes

5. The article is presented in an intelligible fashion and is written in standard English.

Reviewer: Yes

6. The research meets all applicable standards for the ethics of experimentation and research integrity.

Reviewer: Yes

7. The article adheres to appropriate reporting guidelines and community standards for data availability.

Reviewer: Yes

Additional comments.

**Introduction:** The citation on page # 3, line 65 makes it difficult for the reader to follow. I suggest that it should be inserted at the end of the sentence (e.g., urban Shinyanga (reference).

Re-cast the citations on page # 3, line 69-70 (e.g., Sharma et al. 2015; Sabapathy et al. 2012)

Define “ yields” on page # 4, line 78.

A citation at the end of the sentence “….Tanzania is in transition period…..” is missing, on page # 4, line 82-83.

Move the 51.1% just after the words “…At least half (51.1%) of the population…..”, On page # 4, line 85.

Define “ethnographic study” on page # 4,line 92.

It will be beneficial for the readers to know how information from this study will be utilized in the study setting (i.e., who are the primary, secondary, and tertiary beneficiaries). I suggest this should be described after the last sentence on page #4, line 94.

**Study setting and project background:** On page # 5, line 98, kindly provide the approximated population of the Shinyanga Region, and the male: female ratio.

On page # 5, line 102-103, kindly provide (n=) for government dispensaries, Heath clinics, private faith-based Heath centres, and private laboratories. And add a citation to support that data.

On page # 5, line 111-the authors provide the distance-time by cycling….I was curious why only cycling, and not walking/or by a motor vehicle? Any justification for that?

On page #5, line 116-the authors mentioned in town, work and school hours affect….I suggest that providing the time range(e.g, from 7.00 am-4.30 pm) will make it clear for a reader to follow.

**Data collection:** On page #6, line 123, the authors should provide the number of trained Tanzanian researchers who conducted the structured observations (e.g., N=? male: female ratio).

On page # 6, line 140-the authors should provide examples of questions asked for each category (e.g., their testing experiences, their motivation for testing…etc.).

On page # 6, line 143-142, the authors mentioned that participants were asked about the new Treat All policy. How did participant get access to the new policy?

Did the data collection tools pilot test before data collection? I suggest the authors should provide this information and provide the number of participants who participated in the pilot testing and how was the information from the pilot test used.

**Data analysis:** On page #6, line 145, the authors used Qualitative content analysis. I suggest the authors should justify using content analysis instead of other alternative data analysis methods.

On page #6, lines 148-149, the authors describe how the analysis was done. I would like the authors to provide more information for the following: (1) What were the major coding categories?, (2 ). Did the study assessed coder consistency between coders?, (3). Post-coding: did coders examined, compared, and contrast the distribution of themes within and across subgroups, and whether this is reflected in the results section?, (4). Did the in-depth interviews transcripts and direct observation notes analyzed together or separate and later combined?. I suggest authors should describe how they handled this. (5). I did not see mentioned how the study observed reflexivity (i.e., conscious self-reflection to make explicit individual's potential influence on the research process), and steps taken to ensure study rigour. I suggest the author address these two important issues in the qualitative study.

**Results:** A table of participant characteristics would be beneficial to the readers.
